# Improvement in sleep following treatment initiation in newly diagnosed, treatment-naïve patients with Wilson’s disease

**DOI:** 10.1007/s10072-025-08388-0

**Published:** 2025-08-02

**Authors:** Wojciech Jernajczyk, Tomasz Litwin, Agnieszka Antos, Jan P. Bembenek

**Affiliations:** 1 Department of Clinical Neurophysiology, Institute of Psychiatry and Neurology, Sobieskiego 9, Warsaw, 02-957 Poland; 2 Department of Neurology, Stroke Unit and Rehabilitation Subunit, Wolski Hospital, Warsaw, Poland; 3 Second Department of Neurology, Institute of Psychiatry and Neurology, Warsaw, Poland

**Keywords:** Wilson’s disease, Copper, Anti-copper treatment, Sleep effectiveness, Polysomnography

## Abstract

**Introduction:**

Sleep disturbances (SD) are common in chronic liver diseases and neurodegenerative disorders, including Wilson’s disease (WD). However, there is limited evidence regarding improvement in sleep quality following initiation of anti-copper therapy in treatment-naïve WD patients.

**Methods:**

We performed and evaluated video-polysomnography (vPSG) in six newly diagnosed, treatment-naïve WD patients. All patients underwent overnight vPSG before and after initiation of anti-copper treatment.

**Results:**

None of the patients reported subjective SD during routine clinical assessment. Objective improvement in sleep efficiency (SE) was observed in five patients, with increases of 6.4%, 7.4%, 17.1%re, 17.3%, and 45.6%, respectively. In three patients, SE normalized (> 90%) after treatment. One patient demonstrated a decline in SE on follow-up (73.5% vs. 67.4%).

**Conclusions:**

Initiation of anti-copper treatment in newly diagnosed WD patients may lead to improvements in sleep quality, in addition to neurological symptom relief. These preliminary findings warrant confirmation in larger, controlled studies.

## Introduction

Wilson’s disease (WD) is a genetic disorder characterized by pathological copper accumulation in the liver, brain, and other organs. It results from mutations in the *ATP7B* gene, which encodes a copper-transporting ATPase responsible for copper excretion by hepatocytes [1–4]. The disease affects multiple organ systems, including the central nervous system, leading to both neurological and psychiatric symptoms.

Clinical manifestations of WD typically appear between the ages of 5 and 35. In most cases, the initial presentation is hepatic (in 50–60% of patients), ranging in severity from asymptomatic elevations of liver enzymes to hepatosplenomegaly, hepatic steatosis, acute hepatitis, compensated or decompensated liver cirrhosis (up to 50%), and even acute liver failure [1–5]. Neurological symptoms develop in up to 40% of patients during the disease course and include movement disorders such as tremor, dystonia, parkinsonism, chorea, drooling, and secondary gait and posture disturbances, as well as dysphagia and dysarthria [4, 6]. Less common neurological manifestations include peripheral neuropathy, olfactory disturbances, epilepsy, and restless legs syndrome (RLS) [4, 6]. Psychiatric symptoms are also common in WD and may be the initial manifestation in up to 25% of patients [7]. Throughout the illness, psychiatric symptoms are reported in approximately 70–100% of cases [8, 9], and include: cognitive deficits, behavioral and personality disturbances, mood disorders (primarily depression), and other symptoms such as psychosis, anorexia, anxiety, and obsessive-compulsive syndromes [7].

Importantly, WD is a treatable condition if anti-copper therapy (chelating agents or zinc salts) is initiated early and treatment adherence is maintained [8–13]. However, the psychiatric form of WD is often associated with the longest diagnostic delay, which may result in treatment failure and increased mortality [14].

Sleep disturbances (SD) have been increasingly recognized as a global health concern and are associated with a higher risk of cognitive impairment, mood disorders, and cardiovascular disease [15–19]. Several studies have also documented a higher prevalence of SD in patients with WD, though most of these assessments rely on subjective scales rather than objective diagnostic tools [20–26].

Video polysomnography (vPSG) is a comprehensive diagnostic method that monitors brain activity, respiration, heart rate, oxygen saturation, and muscle tone during sleep, allowing for the precise identification of various sleep disorders [27]. Despite its diagnostic potential, only five studies and one case report to date have used vPSG to assess sleep disturbances in WD [21–26]. Till date, only one study evaluated SD in treatment-naïve WD patients [26]. Reported prevalence rates of SD in WD based on vPSG vary from 42 to 80% [22–25], although some studies do not confirm such high rates [24]. Notably, no prior studies have assessed changes in objective sleep parameters following the initiation of anti-copper therapy in treatment-naïve WD patients.

Given the importance of early identification of sleep disturbances in WD and the potential impact of anti-copper treatment, we aimed to evaluate changes in vPSG parameters before and after treatment initiation in newly diagnosed, treatment-naïve patients with Wilson’s disease.

## Methods

### Participants

We prospectively evaluated six adult, treatment-naïve patients with newly diagnosed WD, hospitalized at the Second Department of Neurology, Institute of Psychiatry and Neurology in Warsaw, Poland, between March 2015 and April 2019. Each patient was assessed at two time points: at baseline (before treatment initiation) and at follow-up, approximately two years after the introduction of anti-copper therapy.

The study protocol was approved by the local ethics committee. Inclusion criteria comprised a confirmed diagnosis of WD based on a Leipzig score of ≥ 4 points [28], including *ATP7B* genetic analysis, and the ability to participate in vPSG examination. Written informed consent was obtained from all participants.

### Assessments

Copper metabolism parameters, including serum ceruloplasmin, total serum copper, and 24-hour urinary copper excretion, were evaluated at diagnosis (before anti-copper therapy) and at follow-up, as previously described [29]. Non-ceruloplasmin-bound copper (NCC) was calculated indirectly according to established methodology [29]. All patients underwent brain magnetic resonance imaging (MRI) at baseline and follow-up (2 years later) as part of standard diagnostic procedures. Imaging was performed using a Philips Achieva 1.5T system (Philips Healthcare, Eindhoven, Netherlands). In addition to routine radiological interpretation, all scans were further evaluated using the semiquantitative MRI scale proposed by Dusek et al., which includes assessment of acute toxicity and chronic damage [30]. Additionally we separately checked the brain structures involved in SD: hypothalamus, pineal gland, pons and midbrain in analyzed group of WD patients. Patients were classified as symptomatic or asymptomatic based on clinical presentation, and further subtyped into neurological or hepatic forms depending on predominant symptoms, as previously defined [28]. Neurological assessment was performed at both time points by a trained neurologist (TL) using the Unified Wilson Disease Rating Scale (UWDRS), including Part I (consciousness), Part II (activities of daily living), and Part III (neurological examination) [29]. All patients were also evaluated twice by a psychiatrist and sleep specialist (WJ) and underwent hepatological assessment based on liver biopsy or neuroimaging (MRI, ultrasound) to determine the presence of liver cirrhosis.

Standard EEG was performed in all participants. vPSG was conducted using a Grass Comet (USA) system with a sampling rate of 200 Hz, between 20:00 and 06:00. The vPSG protocol included six EEG leads, chin and anterior tibialis electromyographic channels, electrooculography channels, respiratory and oxygen saturation sensors, electrocardiological sensors, and body position monitoring. All recordings were scored according to the criteria of the American Academy of Sleep Medicine [27]. Each participant underwent vPSG at baseline (before treatment) and at follow-up (2 years after therapy initiation). Patients with other somatic or psychiatric comorbidities or those receiving chronic pharmacotherapy (aside from anti-copper agents) were excluded from vPSG analysis. All patients were treated with either d-penicillamine (DPA) or zinc sulphate (ZS) following WD diagnosis. During the follow-up period, adherence to anti-copper treatment and use of any additional medications (including psychiatric drugs) were recorded, along with the emergence of any new medical conditions.

## Results

Six patients (4 men, 2 women) with newly diagnosed WD were included and underwent vPSG assessments at baseline and follow-up (after 2 years of anti-copper treatment). Demographic and clinical data are summarized in Table 1. The mean age at diagnosis was 35.1 years (range: 17–52), and the average diagnostic delay from symptom onset was 2.7 years.

**Table 1:** Demographic and clinical data of the studied patients

Patient	Parameter	Timepoint	
		Before treatment	Follow-up (2 years later)
1.	Gender	Male	
	Age at WD onset (years)	41	
	Age at WD diagnosis (years)	44	
	Anti-copper tretatment	ZS (180 mg of elementary Zn2+/day)	
	Clinical form of WD	Neurologic form	
	UWDRS part II (points)	1	0
	UWDRS part III (points)	7	2
	Brain MRI: lesions in the pons and/or mesencephalon	yes	yes
	Brain MRI: semiquantitative scale acute toxicity score (points)	1	0
	Brain MRI: semiquantitative scale chronic damage score (points)	3	2
	Serum ceruloplasmin (mg/dl)	19.3	5
	Urinary copper excretion (µg/24 hours)	149	68
	NCC (µg/dl)	17.44	15.5
2.	Gender	Male	
	Age at WD onset (years)	51	
	Age at WD diagnosis (years)	52	
	Anti-copper tretatment	DPA (1000 mg/day)	
	Clinical form of WD	Neurologic form	
	UWDRS part II (points)	2	0
	UWDRS part III (points)	30	11
	Brain MRI: lesions in the pons and/or mesencephalon	yes	yes
	Brain MRI: semiquantitative scale acute toxicity score (points)	3	1
	Brain MRI: semiquantitative scale chronic damage score (points)	6	6
	Serum ceruloplasmin (mg/dl)	3.8	13.3
	Urinary copper excretion (µg/24 hours)	475	220
	NCC (µg/dl)	29.1	14.3
3.	Gender	Male	
	Age at WD onset (years)	18	
	Age at WD diagnosis (years)	23	
	Anti-copper tretatment	DPA (1000 mg/day)	
	Clinical form of WD	Neurologic form	
	UWDRS part II (points)	2	6
	UWDRS part III (points)	18	8
	Brain MRI: lesions in the pons and/or mesencephalon	yes	yes
	Brain MRI: semiquantitative scale acute toxicity score (points)	6	2
	Brain MRI: semiquantitative scale chronic damage score (points)	5	4
	Serum ceruloplasmin (mg/dl)	5.4	1.4
	Urinary copper excretion (µg/24 hours)	165	491
	NCC (µg/dl)	21,5	9,6
4.	Gender	Male	
	Age at WD onset (years)	37	
	Age at WD diagnosis (years)	43	
	Anti-copper tretatment	ZS (180 mg of elementary Zn2+/day)	
	Clinical form of WD	Hepatic form	
	UWDRS part II (points)	0	0
	UWDRS part III (points)	0	0
	Brain MRI: lesions in the pons and/or mesencephalon	yes	yes
	Brain MRI: semiquantitative scale acute toxicity score (points)	0	0
	Brain MRI: semiquantitative scale chronic damage score (points)	0	0
	Serum ceruloplasmin (mg/dl)	16.5	1.5
	Urinary copper excretion (µg/24 hours)	155	80
	NCC (µg/dl)	7.6	14.7
5.	Gender	Female	
	Age at WD onset (years)	16	
	Age at WD diagnosis (years)	17	
	Anti-copper tretatment	DPA (1000 mg/day)	
	Clinical form of WD	Neurologic form	
	UWDRS part II (points)	12	0
	UWDRS part III (points)	21	0
	Brain MRI: lesions in the pons and/or mesencephalon	yes	yes
	Brain MRI: semiquantitative scale acute toxicity score (points)	9	4
	Brain MRI: semiquantitative scale chronic damage score (points)	3	3
	Serum ceruloplasmin (mg/dl)	6.9	1.3
	Urinary copper excretion (µg/24 hours)	136	377.4
	NCC (µg/dl)	29.1	1.2
6.	Gender	Female	
	Age at WD onset (years)	32	
	Age at WD diagnosis (years)	32	
	Anti-copper tretatment	ZS (180 mg of elementary Zn2+/day)	
	Clinical form of WD	Neurologic form	
	UWDRS part II (points)	4	0
	UWDRS part III (points)	5	11
	Brain MRI: lesions in the pons and/or mesencephalon	yes	yes
	Brain MRI: semiquantitative scale acute toxicity score (points)	3	1
	Brain MRI: semiquantitative scale chronic damage score (points)	2	2
	Serum ceruloplasmin (mg/dl)	15.8	3,3
	Urinary copper excretion (µg/24 hours)	56	8
	NCC (µg/dl)	8.0	1.3

*Brain MRI semiquantitative scale* brain magnetic resonance imaging semiquantitative scale (acute toxicity score and chronic damage score); *DPA* d-penicillamine; NCC– non ceruloplasmin bound copper; *UWDRS* Unified Wilson’s Disease Rating Scale (part II activity of daily living, part III detailed neurological assessment); *ZS* - zinc sulphate

Five patients presented with the neurological form of WD, while one patient (no. 4) had the hepatic form. All patients were homozygous for the p.H1069Q mutation in the *ATP7B* gene. Mean UWDRS scores at baseline were 3.5 points (range: 0–12) in Part II and 13.5 points (range: 0–30) in Part III. Brain MRI using the semiquantitative scale yielded a mean acute toxicity score of 3.6 (range: 0–9) and a chronic damage score of 3.1 (range: 0–6). Patients No 1, 4 and 6 were treated before second examination with zinc salts, while patients No 2, 3 and 5 received D-penicillamine. Four patients had cirrhosis confirmed in liver biopsy and by hepatological evaluation. None of the patients showed clinical signs of hepatic encephalopathy, and no characteristic EEG abnormalities suggestive of encephalopathy were detected. None of the patients had abnormal lesions in hypopthalamus and pineal gland in both MRI examinations. Four patients had lesions in pons and 5 patients in mesencephalon, while in follow-up examination lesions were present in pons in 3 patients (in one resolved when compared to initial examinations) and still present in 5 patients in mesencephalon. We did not observe MRI signs of hepatic encephalopathy including T1 hyperintensity in globus pallidus or Diffusion-weighted changes in analysed patients. In five patients (no. 1, 3, 4, 5, and 6), SE improved by 6.4%, 7.4%, 17.1%, 17.3%, and 45.6%, respectively, following treatment. These patients also demonstrated increased total sleep time (by 8.2, 37.5, 64.0, 85.9, and 211.9 min). SE normalized (> 90%) in three patients (no. 1, 4, and 5). REM sleep duration increased at follow-up in four patients (no. 1, 4, 5, and 6): patient 1: 114.4 min (24.9%) vs. 90.5 min (20%); patient 4: 122.5 min (28.1%) vs. 73.5 min (18.4%); patient 5: 127.0 min (28.1%) vs. 85.5 min (23.3%); patient 6: 71.5 min (17.5%) vs. 18.0 min (9.2%). In contrast, REM duration decreased in patient 3, from 104.8 min (30.4%) at baseline to 78 min (19.1%) at follow-up. This patient also had a slight increase in the number of awakenings (15 vs. 13), while the remaining four patients experienced fewer awakenings during follow-up. Patient 2 was the only participant whose sleep parameters slightly deteriorated over the two years. Total sleep time was reduced (327.3 vs. 354.3 min), and sleep efficiency declined (67.4% vs. 73.5%), although REM duration increased (106.3 vs. 66.0 min). Interestingly, this patient demonstrated a sleep architecture frequently seen in narcolepsy (which not necessarily constitute a diagnosis of narcolepsy) in both vPSG assessments, with REM sleep latency of 5 and 19.5 min, REM sleep proportion of 18.6% and 32.5%, a number of awakenings of 35 and 16, and sleep efficiency of 73.3% and 67.5%, respectively. However, no clinical symptoms of narcolepsy were reported, and none were confirmed by the examining sleep specialist. We also did not measure hypocretin levels in those patients.

No patient had periodic limb movements in sleep recorded during vPSG. Additionally, REM without atonia was not present in any of the 12 vPSG recordings in those six patients. Patients did not have history of RLS and dream enactment behavior.

Apnea-Hypopnea Index (AHI) was in normal range (≤ 5) in all patients in both vPSG examinations. Similarly there was reduction in wake after sleep onset (WASO) in in follow-up examination in all patients, except from patient No 2.

All six patients had baseline and follow-up vPSG and none used additional medications in follow-up period that may affect sleep and that could be a reason to exclude from the study.

Detailed vPSG findings are presented in Table 2.

**Table 2:** Polysomnography parameters before and after treatment introduction

Patient		Polysomnography	
	Parameter	Before treatment	Follow-up
1	Total sleep time, min.	451.9	460.1
	Stage 1 N, min. (%TST)	58 (12.8)	46.6 (10.1)
	Stage 2 N, min. (%TST)	247.9 (54.9)	218 (47.4)
	Stage 3 N, min. (%TST)	55.5 (12.3)	81 (17.6)
	Stage REM, min. (%TST)	90.5 (20)	114.5 (24.9)
	Sleep latency, min	9	11
	Latency stage 2, min.	13.5	15.5
	Latency stage REM, min.	74	49
	sleep efficiency, %	88.8%	95.2%
	AHI	2.5	2.3
	WASO	50.5	12
2	Total sleep time, min.%	354.3	327.3
	Stage 1 N, min (%TST)	84.8 (23.9%)	85 (26)
	Stage 2 N, min. (%TST)	167.5 (47.3)	112 (34.2)
	Stage 3 N, min. (%TST)	36 (10.2)	24 (7.3)
	Stage REM, min. (%TST)	66 (18.6)	106.3 (32.5)
	Sleep latency, min.	4.5	11
	Latency stage 2, min.	7	14.5
	Latency stage REM, min.	5	19.5
	sleep efficiency, %	73.5	67.4
	AHI	3.1	2.6
	WASO	123.5	147
3	Total sleep time, min. %	344.9	408.9
	Stage 1 N, min. (%TST)	41 (11.9)	90 (22)
	Stage 2 N, min. (%TST)	107 (31)	180.9 (44.2)
	Stage 3 N, min. (%TST)	92 (26.7)	60 (14.7)
	Stage REM, min. (%TST)	104.8 (30.4)	78 (19.1)
	Sleep latency, min.	18.5	7
	Latency stage 2, min.	7	16.5
	Latency stage REM, min.	54	101
	sleep efficiency, %	71.1	84.8
	AHI	1.4	1.3
	WASO	122	55
4	Total sleep time, min. %	399	436.5
	Stage 1 N, min. (%TST)	41 (10.3)	37.5 (8.6)
	Stage 2 N, min. (%TST)	206 (51.6)	200.5 (45.9)
	Stage 3 N, min. (%TST)	78.8 (19.7)	76 (17.4)
	Stage REM, min. (%TST)	73.5 (18.4)	122.5 (28.1)
	Sleep latency, min.	5.5	6.5
	Latency stage 2, min.	5.5	6.5
	Latency stage REM, min.	77	103
	sleep efficiency, %	82.8	90.2
	AHI	3.4	2.1
	WASO	78.1	45.2
5	Total sleep time, min. %	366.5	452.4
	Stage 1 N, min. (%TST)	86.5 (23.6)	30.5 (6.7)
	Stage 2 N, min. (%TST)	106.5 (29.1)	177.9 (39.3)
	Stage 3 N, min. (%TST)	88 (24)	117 (25.9)
	Stage REM, min. (%TST)	85.5 (23.3)	127 (28.1)
	Sleep latency, min.	18	16.5
	Latency stage 2, min.	18	20.5
	Latency stage REM, min.	180	88.5
	sleep efficiency, %	76	93.1
	AHI	3.4	1.9
	WASO	110.5	18.5
6	Total sleep time, min. %	195.1	408
	Stage 1 N, min. (%TST)	94.6 (48.5)	81.5 (20)
	Stage 2 N, min. (%TST)	26 (13.3)	138.5 (33.9)
	Stage 3 N, min. (%TST)	56.5 (29)	116.5 (28.6)
	Stage REM, min. (%TST)	18 (9.2)	71.5 (17.5)
	Sleep latency, min.	14.5	120
	Latency stage 2, min.	61	236
	Latency stage REM, min.	319	77
	sleep efficiency, %	39.3	84.9
	AHI	4.1	1.8
	WASO	298	52.4

*Min.* minutes; *EEG* electroencephalography; *TST* total sleep time; *REM* rapid eye movement, *AHI* Apnea-Hypopnea Index; *WASO* wake after sleep onset

Sleep efficiency is the ratio of sleep time to time spent in bed expressed in %

**Fig. 1:**
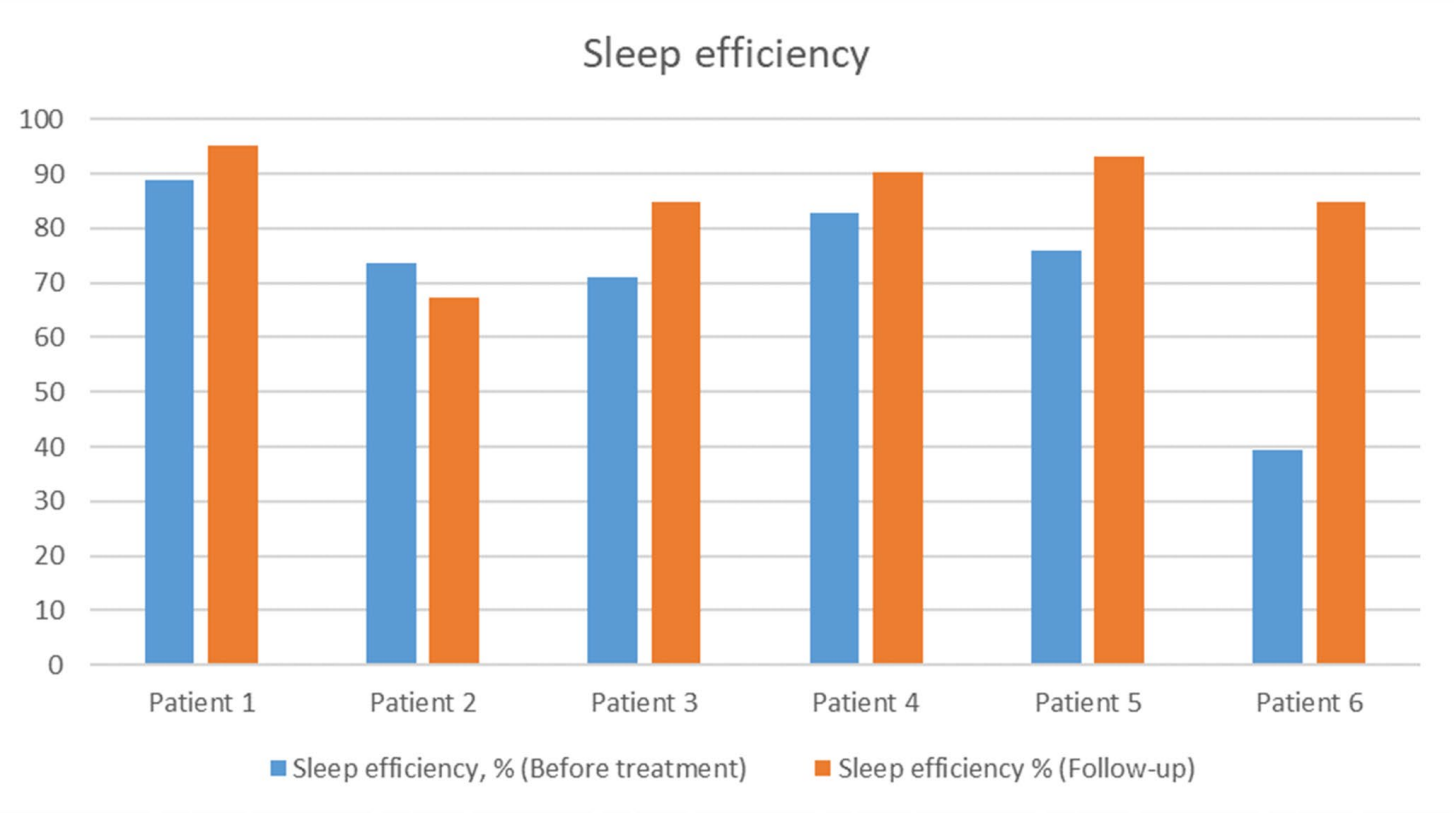
Changes in sleep efficiency before and after treatment introduction

**Fig. 2:**
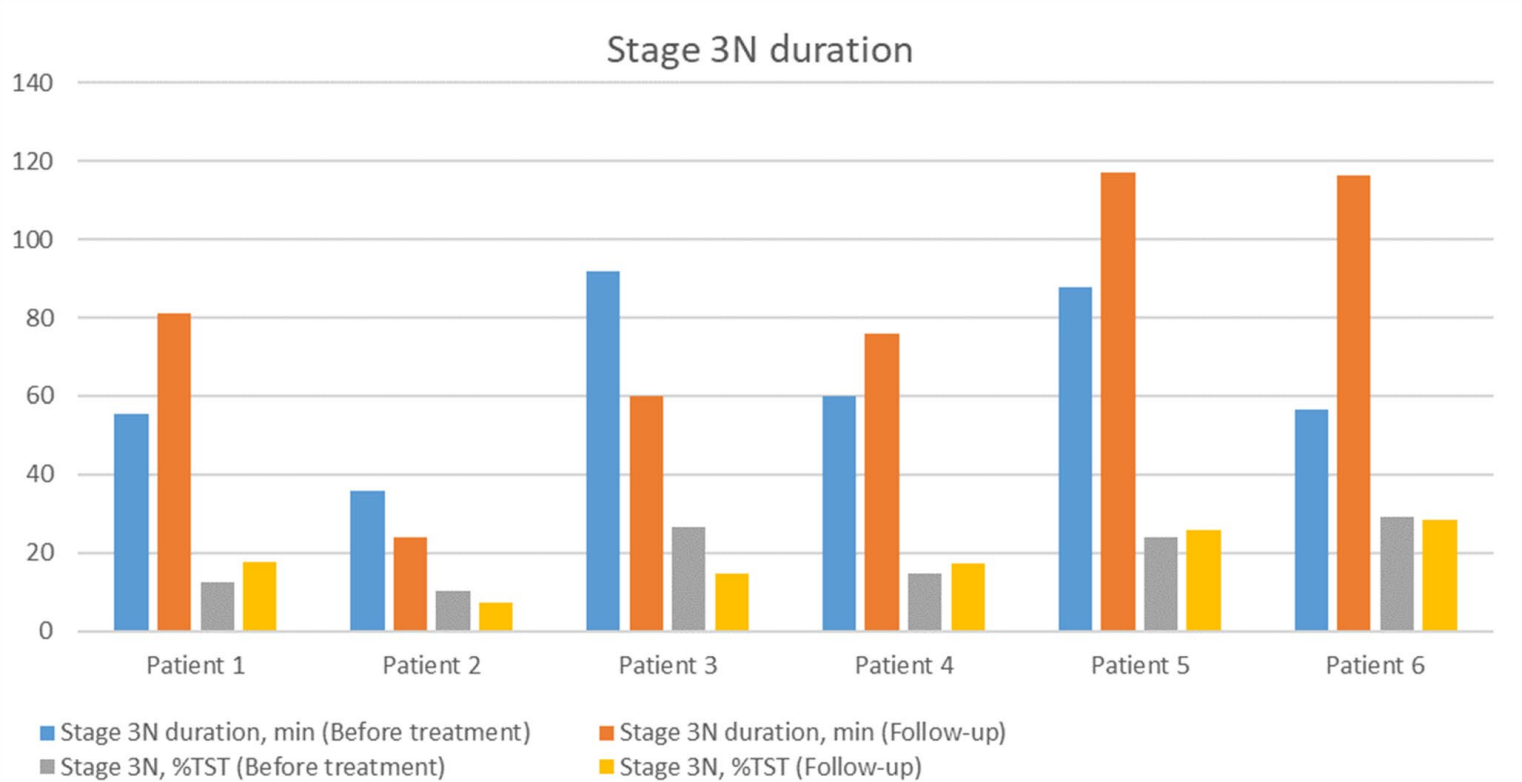
Changes in stage 3 N duration before and after treatment introduction

**Fig. 3:**
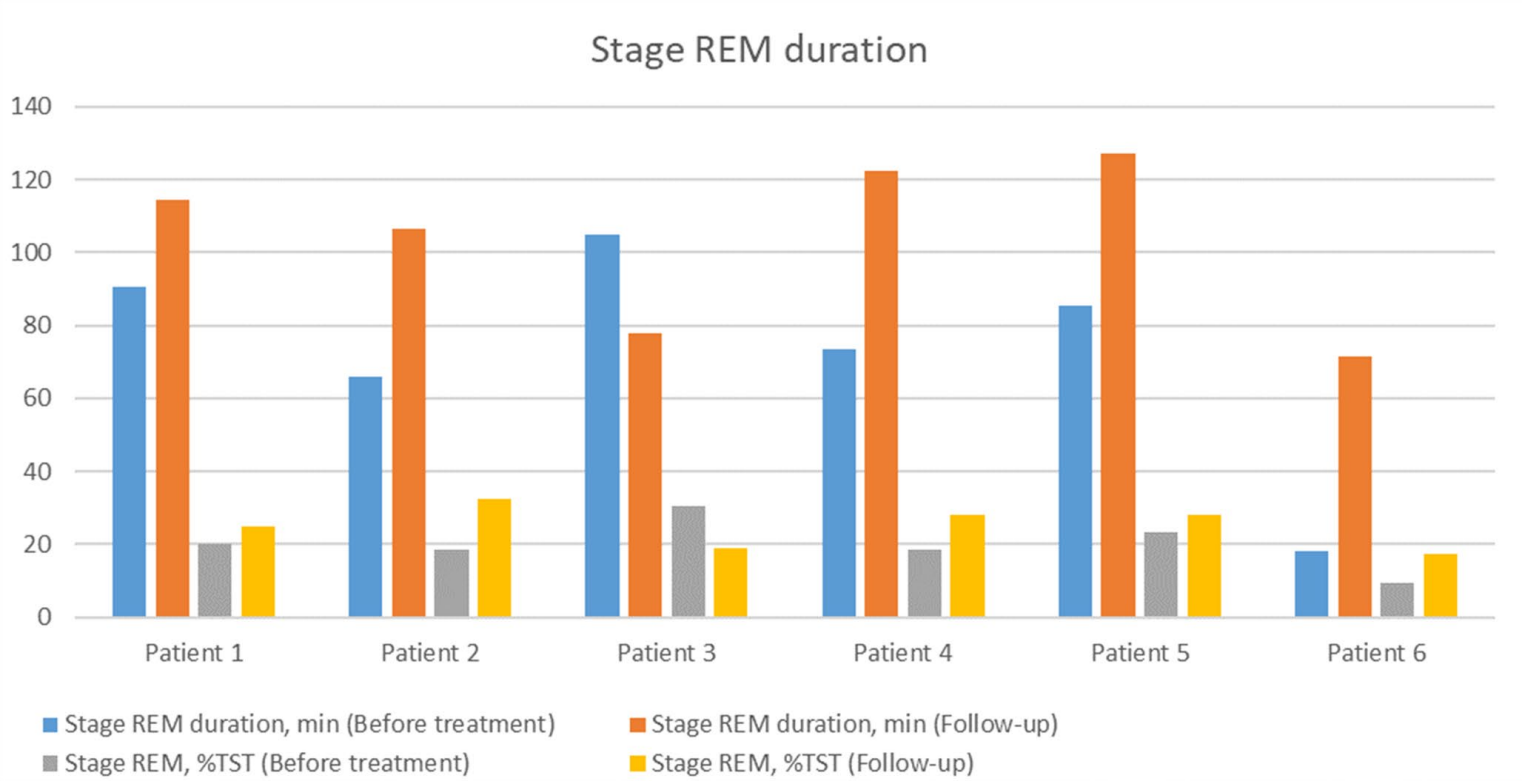
Changes in stage REM duration before and after treatment introduction

Baseline and follow-up sleep efficiency, N3 and REM duration are presented in Figs. 1, 2 and 3.

## Discussion

This study is the first to demonstrate that the initiation of anti-copper treatment in newly diagnosed, treatment-naïve WD patients may lead to improvements in sleep parameters and sleep quality, as assessed objectively using vPSG. Following treatment introduction, five out of six patients showed improved sleep efficiency, while in one case no improvement was observed. Notably, this patient displayed a vPSG pattern characteristic of narcolepsy, which may explain the absence of treatment effect. However, a reduced REM latency and a high number of arousals do not necessarily constitute a diagnosis of narcolepsy.

Sleep disturbances in WD are reported in 42–80% of patients, including frequent nocturnal awakenings, poor sleep quality (RLS), delayed morning wake-up, daytime sleepiness, difficulties initiating sleep, and restless leg syndrome, with patients often reporting unrefreshing sleep [21–26, 31–35]. Despite their frequency, sleep disorders in WD remain underrecognized and insufficiently studied. Previous publications have documented sleep problems based primarily on scales (e.g., Athens Insomnia Scale, Epworth Sleepiness Scale), which, in contrast to vPSG, offer limited diagnostic precision and no follow-up data. Although RLS is often recorded during vPSG in extrapyramidal disorders, we did not record it in our cohort. However, small number of patients and short period of WD symptoms may at least partially explain this.

Several mechanisms may contribute to sleep disturbances in WD: (1) degeneration or dysfunction of sleep-regulatory pathways due to pathological copper accumulation; (2) psychiatric comorbidities such as anxiety or psychosis; (3) motor symptoms including tremor, dystonia, akinetic syndrome with pain, and RLS; (4) iatrogenic effects, particularly of psychiatric medications; (5) pineal night-specific ATP-ase (PINA) dysfunction affecting melatonin secretion; and (6) liver damage leading to ammonia or manganese intoxication and neurotransmitter imbalances [6, 26, 36].

In WD copper is deposited and damages the reticular formation (including mesencephalon and pons) because there are morphological damages typical for WD which was confirmed in our patients. However, in our group we did not confirm the presence of lesions in hypothalamus and pineal gland which are also involved in SD. However, we know from the literature that copper is deposited in all tissues, so it is not possible to say unequivocally that other brain structures in WD are not also affected at the metabolic level [1, 4].

As previously discussed, the main clinical forms of WD include hepatic and neurological presentation [1–4]. Given that WD symptoms result from systemic copper overload, they can be reversed with appropriate anti-copper therapy. Improvements in liver function typically occur within six months, while neurological recovery may take up to two years [37, 38]. We confirmed sleep parameters improvement after 2 years since treatment initiation however, it is possible that improvement could be observed earlier. Repeated vPSG tests would help determine the time at which improvement occurs. Key factors influencing treatment outcomes include early diagnosis and appropriately administered anti-copper therapy, with attention to patient adherence, adverse drug reactions, and especially cautious use of psychiatric medications due to their potential to worsen neurological status [1, 37–40].

Although the role of copper in psychiatric disorders (particularly cognitive dysfunction, psychosis, and depression) has been extensively investigated, limited data exist on its association with sleep disturbances [41, 42]. In the general population, sleep disorders are known to precede and exacerbate psychiatric conditions such as anxiety and mood disorders, which may worsen WD prognosis through increased mortality and heightened risk of neurological deterioration, especially when antipsychotics are used [39, 43, 44]. Our findings suggest that early identification and treatment of sleep disorders in WD may contribute to better clinical outcomes. In our cohort, all patients demonstrated clinical improvement, normalization of liver function tests, better results of brain MRI, improvement in copper metabolism markers, and enhanced sleep parameters on vPSG (Tables 1 and 2). These observations support the hypothesis that anti-copper treatment reduces direct and indirect brain toxicity (e.g., oxidative stress) and mitigates hepatic-related mechanisms such as increased ammonia or manganese levels or false neurotransmitter production, thereby normalizing sleep [44–47]. This also contributed to the improvement of sleep efficiency and increased stage REM duration in our patients. Importantly, none of the patients with baseline sleep disturbances experienced neurological or psychiatric deterioration during follow-up. Moreover, no patient required additional psychiatric medications such as antidepressants or neuroleptics; improvement was achieved solely through anti-copper therapy.

The use of vPSG in WD may also provide added clinical value beyond sleep evaluation. Identifying specific patterns, such as sleep apnea, insomnia, RLS, or excessive daytime sleepiness, can help tailor individual management strategies, for instance, introducing Continuous Positive Airway Pressure (CPAP) therapy [45–47].

To date, no prospective studies have applied vPSG in the assessment of sleep disorders in WD, aside from a case report by Firneisz et al., who described a patient with WD and hypersomnia documented by vPSG, with complete recovery following 8–10 months of DPA therapy [42]. This finding further supports our observations as we observed improvement in vPSG in five out of six patients in follow-up examinations.

Finally, experimental studies in animal models of WD have suggested that melatonin may offer a supplementary anti-copper effect, in addition to its antioxidant and sleep-regulating properties [48–50]. Given its wide clinical use and safety profile, we propose that melatonin should be evaluated in future clinical trials as an adjuvant to anti-copper therapy in WD, potentially benefiting both copper metabolism and sleep quality. However, we did not test melatonin levels in our cohort.

### Study limitations

The main limitation of our study is the small number of participants, which makes it difficult to draw firm conclusions and limits the analysis to descriptive data. Nonetheless, sleep improvement was observed in the majority of WD patients (5 out of 6 cases; 83%). It is important to emphasize, however, WD is a rare condition, and even international, multicenter clinical trials– particularly those involving newly diagnosed, drug-naïve patients– typically include limited sample sizes (approximately 30–40 participants). Another limitation is that we did not evaluate certain biochemical and neuroimaging parameters potentially relevant to the pathophysiology of sleep disturbances in WD. These include serum ammonia, neurotransmitters such as dopamine and noradrenaline, melatonin and hypocretin levels, and specific brain structures involved in sleep regulation (e.g. the pineal gland) [20, 38]. Finally, we did not use questionnaires assessing sleep quality and mood disorders.

## Conclusions

This prospective study is the first to demonstrate that introducing anti-copper treatment in newly diagnosed patients with WD may improve objectively assessed sleep parameters, including sleep efficiency, total sleep time, and REM sleep, as measured by vPSG. vPSG can be an important diagnostic tool in patients with WD, especially when they present with SD. It enables the identification of specific sleep-related disorders and may reflect the extent or progression of neurological involvement. Given that SD significantly affect quality of life and may precede or predict psychiatric and neurodegenerative complications, routine screening for sleep disorders in WD patients should be considered. Importantly, our results indicate that effective anti-copper therapy alone may lead to improvement or even normalization of SD in newly diagnosed patients, without the need for additional psychotropic medications. Based on the proposed pathophysiological mechanisms, future research should also explore the potential role of melatonin as an adjunct to anti-copper therapy in this population. Our study supports the need for further prospective investigations involving larger cohorts to validate these preliminary findings.
